# Evaluation of Brain SPECT with ^99m^Tc-TRODAT-1 in the Differential Diagnosis of Parkinsonism

**DOI:** 10.1155/2022/1746540

**Published:** 2022-03-08

**Authors:** Giorgio Fabiani, Carlos Henrique Ferreira Camargo, Raul Martins Filho, Gabriel Sampaio Froehner, Hélio Afonso Ghizoni Teive

**Affiliations:** ^1^Neurological Diseases Group, Post-Graduate Program of Internal Medicine, Department of Internal Medicine, Hospital de Clínicas, Federal University of Paraná, Curitiba, Paraná, Brazil; ^2^Movement Disorders Unit, Neurology Service, Department of Internal Medicine, Hospital de Clínicas, Federal University of Paraná, Curitiba, Paraná, Brazil; ^3^Neurology Service, Hospital Angelina Caron, Campina Grande do Sul, Curitiba, Paraná, Brazil; ^4^Centro de Medicina Nuclear do Paraná, CETAC—Center for Imaging Diagnosis, Curitiba, Paraná, Brazil

## Abstract

**Introduction:**

Brain SPECT with ^99m^Tc-TRODAT-1 (SPECT-TRODAT) may be a useful tool in the differential diagnosis of Parkinsonism.

**Objective:**

To compare results of SPECT-TRODAT with clinical findings in patients with Parkinsonism.

**Methods:**

We evaluated 153 outpatients. SPECT-TRODAT results were visually analyzed into normal, abnormal, symmetric, and asymmetric, and according to the degree of impairment into mild, moderate, marked, and severe (1–4).

**Results:**

A direct relationship was found between motor scores severity (MDS-UPDRS-III) and SPECT-TRODAT-reduced binding in general, in the group of patients with synucleinopathies (rho = 0.258, *p*=0.005), especially in patients with Parkinson's disease (rho = 0.204, *p*=0.049). Changes in SPECT-TRODAT had high correspondence with symmetry in all Parkinsonism. When comparing groups to the correspondence predominantly bilateral or unilateral impairment in SPECT, there was a difference between patients with SNP (*p*=0.041) and between this group and patients with secondary Parkinsonism (SP) (*p* < 0.0001). It was handy in differentiating drug-induced Parkinsonism from synucleinopathies. In the group of drug-induced Parkinsonism, younger people were the ones who showed the most significant reductions in radiotracer uptake. In this group, nonmotor signs resulted in examinations with more significant reductions in radiotracer uptake. When the scans without alterations and those that did not correspond to the symmetry were considered negative, SPECT-TRODAT's accuracy and specificity to differentiate PD from other forms of Parkinsonism were low. There was an inverse correlation between the severity of the SPECT-TRODAT result and the absence of nonmotor signs in patients with drug-induced Parkinsonism.

**Conclusion:**

The authors concluded that the SPECT with 99mTc-TRODAT-1 was mainly useful in differentiating between synucleinopathies and secondary Parkinsonism.

## 1. Introduction

It is estimated that 1% to 3% of the world population over 65 years of age has Parkinson's disease (PD) [1, 2]. A Brazilian study that evaluated 1528 patients showed PD as the most common diagnosis representing 74.7% of the patients with Parkinsonism, followed by drug-induced Parkinsonism (DIP) in 7.9%, vascular Parkinsonism (VP) in 3.9%, other neurodegenerative diseases in 10%, and rare sporadic causes divided into genetic, infectious, and other, which totaled 3.5% [3].

Parkinsonism includes, in addition to PD, degenerative diseases such as progressive supranuclear palsy (PSP), multiple system atrophy (MSA), Lewy body dementia (LBD), cortico-basal degeneration (CBD), and secondary Parkinsonism (SP) such as DIP and VP [1, 3, 4].

A particular group of neurodegenerative diseases is characterized by abnormal synuclein accumulation, termed alpha-synucleinopathies (SNP); this group includes PD, LBD, and MSA. Alpha-synuclein is a protein that forms Lewy bodies (LBs). Various therapeutic targets, including immunotherapies targeting synuclein, have been studied [5–8]. In tauopathies (TP), tau protein (P-tau) accumulates. The primary TPs are Alzheimer's disease (AD), PSP, CBD, argyrophilic grain disease, Pick's disease, and frontotemporal degeneration with Parkinson's disease (FTD-P) associated with chromosome 17. TP pathology involves both neurons and glial cells. Although TP and SNP are viewed and classified as pathologically distinct clinical entities, cases may occur with fusion or overlap of both, making it challenging to classify them [5].

The importance of this study can be expressed by the high incidence of Parkinsonism and the need for accurate and rapid diagnosis. The differential diagnosis of Parkinsonism mainly considers clinical findings [1, 3, 4, 6, 7]. However, imaging tests and other markers may be essential tools for a quick and correct diagnosis. Since the 80s, with the emergence of PET (positron emission tomography) and SPECT (single-photon emission tomography) functional radiotracers, it became possible to analyze the integrity of dopaminergic systems *in vivo*, which has helped in the differential diagnosis between PD and other Parkinsonism [8–15].

This study aimed to compare the results of the brain SPECT examination with the radiotracer ^99m^Tc-TRODAT-1 (SPECT-TRODAT) in outpatients with Parkinsonism by correlating and comparing them with symptom severity and the presence of nonmotor signs (NMS).

## 2. Material and Methods

### 2.1. Patient Selection

We evaluated 350 medical records of patients followed at the neurology services (Parkinson's disease and movement disorders outpatient clinic at Hospital Angelina Caron, Policlínica de Araucária, and a private clinic (GF)) from January 2015 to June 2020. We included patients examined by the same neurologist specialized in movement disorders (GF), who had presented for at least three consultations and had their medical records complete (neurological examination and investigation scales completed). The diagnostic criteria should correspond to one of the following disorders: PD [6], MSA [16], CBD [17], LBD [18], PSP [19], VP [20], or DIP [21]. The patients must have performed their SPECT-TRODAT in the same imaging laboratory (CMN Unit of CETAC Group, in Curitiba) and reports provided by the same nuclear physician (RMF). Divergences were evaluated by two other specialists in movement disorders (CHC and HT). Of the 350 initial records, 197 were excluded; 120 were excluded due to the lack of any essential question in their records or the lack of any of the assessment scales duly filled out; 48 were excluded due to essential tremor; 29 presented exams from other services or reports provided by another nuclear physician. Finally, 153 records were selected for participation in this review study.

### 2.2. Ethical Aspects

The study project was submitted to the Ethics Committee of the Angelina Caron Hospital (CEP-HAC) on 28/01/2019 and was approved on 24/02/2019 under CAAE protocol number: 07802119.3.0000.5226.

### 2.3. Clinical Assessment

Demographic data, clinical history, disease progression, use of medications, complementary exams, and family history were collected using a standardized questionnaire. The neurological physical examination was performed focusing on movement disorders with applying the MDS-UPDRS-III (Movement Disorders Society-Unified Parkinson's Disease Rating Scale-Part III) scale [22]. The motor symptoms part of the MDS-UPDRS scale consists of 18 items. Each item scores from 0 to 4, with the minimum being 0 and the maximum being 132. The Hoehn and Yahr Scale [23] was used to assess the motor severity of the disease. It is divided into levels of severity from 1 to 5. The clinical presentation was classified according to the predominance of bradykinesia or tremor [24].

The Questionnaire for Screening REM Sleep Behavior Disorder (QT-RSB) was used [25]. Its score ranges from 0 to 13 points; studies have shown that sums above 3.5 have a sensitivity of 84%. For constipation, the 2010 Rome III criteria were considered [26]. As we did not use any certified smell test, we excluded our results from it. The restless legs syndrome/Willis–Ekbom disease diagnostic criteria [27] questionnaires were used to evaluate the RLS symptoms.

### 2.4. SPECT-TRODAT Examination

#### 2.4.1. Patient Preparation

All patients were instructed to stop any medication that could interfere with the evaluation of the examination (e.g., anti-Parkinsonian drugs) and remained immobile during the entire course of the examination in a gamma chamber with two GE Millennium ME detectors (Milwaukee, Minnesota, USA).

#### 2.4.2. Radiotracer Preparation

The route of administration of the radiopharmaceutical ^99m^Tc-TRODAT-1 (Institute of Nuclear Energy Research, Atomic Energy Council Executive, Yuan, Taiwan, imported and distributed in Brazil by MJM Produtos Farmacêuticos e de Radioproteção Ltda. CNPJ: 04.891.262/0001-44, Porto Alegre/RS) was intravenous (IV). The recommended dose for scintigraphy of brain processes in adult patients weighing 70 kg is 814 to 1036 MBq (22 to 28 mCi). In patients weighing less than 70 kg, the dose was adjusted. The TRODAT-1 kit was labelled with technetium-99m; each case was labelled with a maximum activity of 44 mCi derived from freshly generated eluate. The maximum volume taken from the generator was less than 5 mL; the material was added to the remaining 0.9% saline solution in a sterile vial, homogenized until complete dilution, and incubated for 30 minutes in a water bath at 100°C.

After incubation, the material was left to cool at room temperature in an appropriate container for 5 minutes. Before administration, visual appearance, radiochemical purity, and pH were checked. The dose activity was administered between 814 and 1030 MBq (22–28 mCi) in an approximate volume of 2 ml for patients with peripheral venous access.

### 2.5. Image Acquisition and Analysis

For system calibration, the imaging laboratory used phantoms and the routine calibrations procedures recommended by the GE and Brazilian National Nuclear Energy Commission (CNEN). Images were acquired and reconstructed in the transverse, coronal, and sagittal planes 4 hours after venous injection of ^99m^Tc-TRODAT. Circular stepwise orbits were used, and a shot power window of 140 ± 15 keys and 128 × 128 with a diameter and rotation degree 360 was used. The acquisition time per projection was 30 seconds and zoom 1.0.

Butterworth-filtered back-projection reconstruction algorithm was used. The images were obtained after processing with 3.39 mm of thickness. Visual quantification was used considering the intensity of the radiopharmaceutical uptake with the background radiation and the occipital cortex.

**Figure 1:**
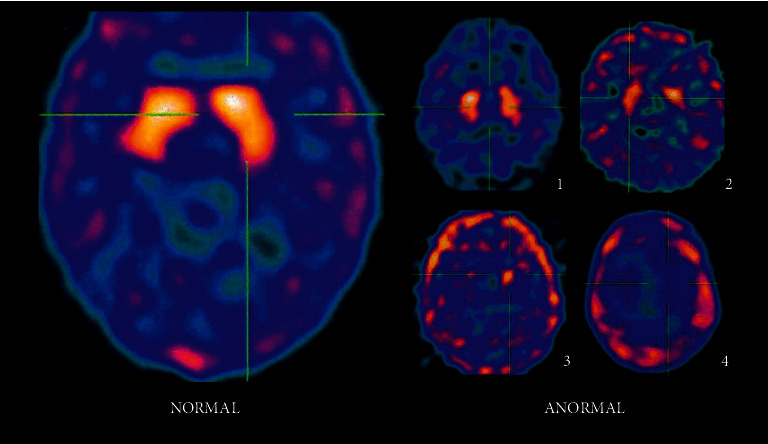
Degrees of involvement according to radiopharmaceutical uptake in cerebral SPECT with ^99m^Tc-TRODAT-1.

We used a visual, qualitative evaluation based on the same principles of Catafau et al. [11] to interpret the images. The results are divided between normal, abnormal, symmetrical, and asymmetrical, with mild, moderate, marked, and severe (1–4) degrees of impairment (Figure 1).

### 2.6. Statistical Analysis

Results were presented as means, medians, minimum and maximum values, and standard deviations (quantitative variables) or as frequencies and percentages (categorical variables). Student's *t*-test or the nonparametric Wilcoxon–Mann–Whitney test was used to compare two groups in terms of quantitative variables. One-way ANOVA or the nonparametric Kruskal–Wallis test compared more than two groups in terms of quantitative variables. The Kolmogorov–Smirnov test assessed the normality of the variables. The chi-square test or Fisher's exact test was used to compare the groups in terms of categorical variables. To assess the association between two quantitative variables, Pearson's or Spearman's correlation coefficient was estimated, depending on the normality of the variables. The ROC curve was used to calculate SPECT-TRODAT's accuracy, sensitivity, and specificity for PD and other Parkinsonism differentiation. Values of *p* < 0.05 indicated statistical significance. Data were analyzed using IBM SPSS Statistics v.20.0 software. Armonk, NY: IBM Corp. 5, Minitab 16 and Excel Office 2010.

## 3. Results

Among the 153 patients with Parkinsonism, most had SNPs (116, 75.8%) divided into 93 (60.8%) with PD, 16 (10.45%) with LBD, and 7 (4.57%) with MSA, all MSA with Parkinsonian form (MSA-P). Five patients (3.27%) were with TP, 2 (1.3%) with PSP, and 3 (1.96%) with CBD. Thirty-two cases (20.91%) were with SP, 19 (12.41%) with DIP, and 13 (8.49%) with VP.

Table 1 summarizes the main clinical and epidemiological data of the patients with Parkinsonism. The patients with DIP had a higher mean age, and there was a statistical difference between the PD patients (*p*=0.012) and the group of patients with SNP (*p*=0.017). There was also a difference between the cases of SP and PD in relation to the age of disease onset with SP patients starting symptoms later, VP (*p*=0.009) and DIP (*p*=0.004). The duration of illness was longer among patients with SNP than patients with DIP (*p*=0.002) and VP (*p*=0.06).

Differences occurred between clinical presentations according to the type of Parkinsonism. Among the SNP, patients with PD presented predominantly with the tremor-bradykinesia (mixed or classic) form, while patients with LBD and MSA with the akinetic-rigid form (*p*=0.0002). The patients with VP presented exclusively with the akinetic-rigid form; therefore, there was a statistical difference with the SNP (*p* < 0.0001). Patients with VP were also all akinetic-rigid with a statistical difference for those with SNP (*p*=0.0002) and PD (*p*=0.0001). The patients with DIP had a similar clinical presentation to the patients with PD. There was a difference for these patients with SNP cases (*p*=0.002).

Regarding the severity of motor signs, the mean UPDRS-III scores in the patients with SNP were 33.1 ± 14.76 points versus 55.85 ± 12.7 in those with VP (*p*=0.0004). A statistical difference was found between these two groups concerning the H&Y scale (*p*=0.001). As it is a chart review study, we included patients in all H&Y stages, with a minority in stage 4. Patients with SP were also in more advanced stages of the disease than patients with SNP (Table 1).

In 90.3% of PD patients, at least one NMS was found. The most prevalent NMS was the REM sleep behavior disorder (RSBD). RLS was the least prevalent NMS occurring in 34.1% of PD patients. There was a statistical difference between the cases of SNP and VP (*p*=0.020) (Table 1).

Regarding the brain SPECT-TRODAT results, only patients with SP had normal exams; 42.1% in the DIP group and 33% in the VP group. A statistical difference was found regarding the percentage of altered exams between the patients with PS and those with SNPs, including with PD (*p* < 0.0001) (Table 2). Regarding the symmetry of changes in the basal ganglia on SPECT-TRODAT, the result was asymmetric in 82.3% of patients with PD. In comparison, 60% of the altered examinations in patients with VP were symmetric. No relationship was observed between the severity of the SPECT-TRODAT alteration and the dominant phenotype (Table 2).

**Figure 2:**
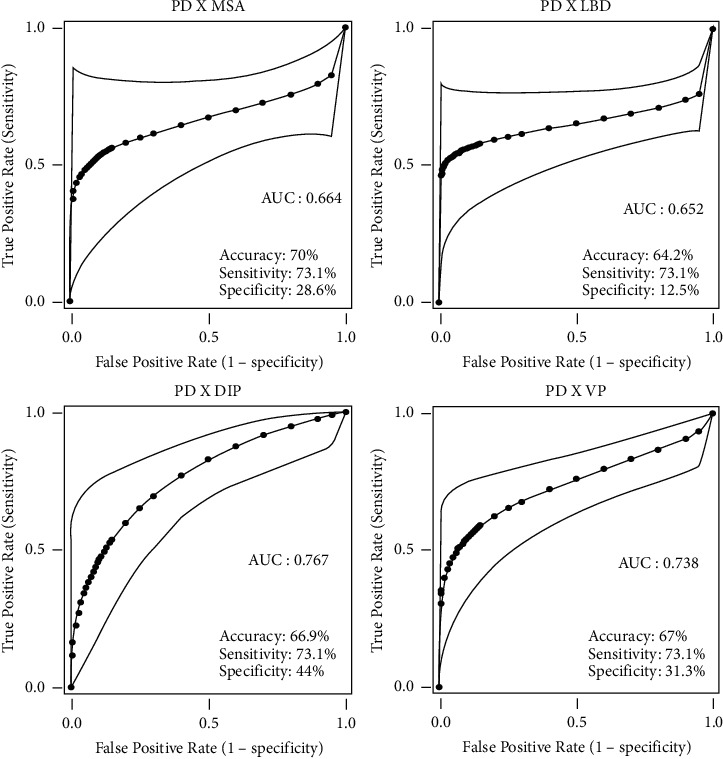
Comparison of SPECT-TRODAT results among patients with Parkinson's disease and other Parkinsonisms^*∗*^. ^*∗*^Normal results and those that did not correspond to the clinical picture (symmetry) were considered negative. For the calculations, unilateral and bilateral results were considered different. PD = Parkinson's disease, MSA = multisystem atrophy, LBD = Lewy body dementia, DIP = drug-induced Parkinsonism, VP = vascular Parkinsonism.

The changes in SPECT-TRODAT corresponded to the motor alterations (bilateral and symmetrical or contralateral) in 68 (73.18%) patients with PD, 5 (71.42%) with MSA, 11 (68.75%) with LBD, 2 (100%) with PSP, 2 (66.6%) with CBD, 17 (89.4%) with DIP, and 11 (84.6%) with VP. The level of matching was high for all Parkinsonism groups, with no statistical difference. When comparing groups regarding the correspondence of the SPECT with predominantly bilateral or unilateral impairment, there was a difference between patients with SNP (*p*=0.041) and between this group and patients with DIP (*p* < 0.0001) and VP (*p* < 0.0001), which showed higher correspondences when the cases had a bilateral clinical presentation. There were statistical differences between patients with DIP (*p* < 0.0001) and VP (*p* < 0.0001), also with the patients with PD, whose presentation was predominantly unilateral (Table 2). When considering the normal scans, the scans that did not match the clinical symmetry as negative, and the scans in which the changes matched the clinical symmetry as positive, the accuracy and specificity for differentiating PD from other Parkinsonism by SPECT-TRODAT were low. The highest specificity was found for differentiation of patients with DIP, 44% (Figure 2).

The age at disease onset had an inverse correlation with the severity of the SPECT-TRODAT result in patients with DIP (rho = −0.539, *p*=0.017). SPECT-TRODAT severity directly correlated with the severity of motor changes measured by MDS-UPDRS-III in the group of patients with SNP (rho = 0.258, *p*=0.005) and the patients with PD (rho = 0.204, *p*=0.049). There was an inverse correlation between the severity of the SPECT-TRODAT result and the absence of NMS in the patients with DIP (rho = –0.451, *p*=0.052).

## 4. Discussion

Imaging with DAT/SPECT is increasingly present in evaluations of neurological patients functioning as highly specific and reliable imaging biomarkers in diagnosing and following neurodegenerative diseases, including PD, other SNPs, VP, and DIP [7–15, 28–35]. Seeking an earlier diagnosis, preferably in the premotor phase of PD, the valorization of NMS as clinical biomarkers is occurring, especially in conjunction with imaging, genetic, or biochemical biomarkers [1, 29]. The ease in analyzing the images obtained by SPECT-TRODAT, by visual inspection, allows a well-trained neurologist to correlate them with the neurological examination findings [8, 9, 11, 14].

In our study, the largest group was formed by patients with SNP (75.8%), with a predominance of PD (60.8%), and the second by patients with SP (20.91%). Except for the higher number of women, our results were compatible with those found in the literature, even when compared to the results of studies stratified by age range for onset of symptoms [13]. Patients with PD had a mean age of 65.09 ± 12.5 years (32–88.9) and the VP 75.62 ± 8.36. These results were similar to those found by Pagano et al. [13] with 61.6 ± 9.7 for PD and by Tzen et al. [15] with 70 ± 7.5 for VP. We believe that these differences in age and gender between the Parkinsonism did not influence the differences obtained in the SPECT-TRODAT results because most studies with PET-CT and SPECT have not shown statistically significant differences between changes in DAT uptake concerning sex [13, 14, 28–32]. Moreover, DAT/SPECT uptake rates in healthy patients are stable between 30 and 70 years of age, allowing someone around 70 years of age to have SPECT-TRODAT results similar to another 30-year old [14].

We found no correlation between the duration of disease and the severity of changes on SPECT-TRODAT scans in Parkinsonism. Differently, Fang et al. [28] and Benamer et al. [9] demonstrated a strong correlation between the reduction in striatal radiotracer uptake and the duration of PD. Regarding the age at onset, we found no correlation with SPECT-TRODAT results in PD patients. Sasannezhad et al. [29] found no differences between patients with PD and those with early-onset PD. In both forms of presentation, there was a significant reduction in DAT uptake on the SPECT-TRODAT. In contrast, Pagano et al. [13] demonstrated that older age at onset was associated with more severe motor and nonmotor phenotypes and more significant dopaminergic dysfunction on DaTSCAN. We presented a correlation of less reduced SPECT-TRODAT uptake and the lower the age at disease onset in patients with DIP (rho = −0.539, *p*=0.017) that had not been reported previously.

A direct relationship was found between severity of DAT reduction in SPECT-TRODAT and motor changes, especially in SNP (rho = 0.258 and *p*=0.005) and in PD group (rho = 0.204, *p*=0.049). Most published studies confirm this correlation between severity in striatal DAT reduction and higher UPDRS-III and H&Y scale scores [9, 1, 13, 14, 28–30].

Only patients with SP had normal SPECT-TRODAT scans. Yomtoob et al. [10] were able to detail the main changes in 51 selected cases of DIP. In 36 patients (70.6%), normal scans were found against only 15 (29.4%) with alterations on SPECT-TRODAT but without specifying them. Those with more than two motor symptoms (tremor, rigidity, bradykinesia, or postural instability) were more likely to have altered scans (63.89% vs. 93.33%, *p*=0.04). Most studies of SPECT-TRODAT in patients with PD correlate the imaging findings or worse side of the exam with the contralateral of the symptoms, or worse side of the patient, with results similar to ours, 73.18% [9, 11, 13, 14]. In VP and DIP, we found a high correspondence between bilateral symptoms and bilateral findings of alterations in SPECT, a fact not discussed in studies already published [10–12, 15, 31, 33, 34]. However, we had SPECT results in all types of Parkinsonism that did not correspond to the patients' clinical findings. When we used the correspondence of the alteration to clinical alterations as a criterion for a positive SPECT-TRODAT result, our SPECT-TRODAT accuracy and specificity rates for differentiating PD from other Parkinsonism were low. One of the first SPECT studies with a good number of patients analyzed was Catafau et al. [11]. The authors demonstrated 100% sensitivity for patients with presynaptic Parkinsonism and 94% for others. However, the study differentiated only abnormal from regular scans. Weng et al. [32] demonstrated 100% sensitivity and specificity in discriminating PD patients from healthy subjects. The study by Pitella [17] demonstrated that qualitative evaluation of SPECT-TRODAT has a sensitivity of 90.91%, specificity of 91.3%, and accuracy of 92.54% for PD.

Tzen et al. [15] compared patients with PD versus controls and VP and observed significant asymmetry in PD patients but not in VP patients (*p* < 0.01). The patients in the VP group tended to have more excellent symmetry of the examinations. The authors concluded that ^99m^Tc-TRODAT-1 SPECT was a reliable method to differentiate between VP and PD. Other authors have also demonstrated the usefulness of brain SPECT in differentiating between PD and VP [11, 12, 15, 31, 34]. The difference between the correspondence of bilateral symptoms of the groups of SP and scans showing more significant symmetries than the group of SNP reflects the different pathophysiological mechanisms involved. In PD, the impairment from the beginning occurs asymmetrically; whereas, in DIP and VP, pathophysiologically different from PD, the impairment would occur symmetrically and homogeneously. In patients with DIP, it would be necessary to have a block of around 80% of the presynaptic neurons starting at 40 years of age [31].

The changes observed in our study would have prognostic and therapeutic implications. A reasonable prognosis of recovery is expected in the DIP with normal SPECT-TRODAT after removing the causative agent. On the other hand, DIP with strongly asymmetric SPECT-TRODAT results may indicate latent PD and symptoms precipitated by the use of dopamine reuptake blocker drugs [36]. In the VP group, an abnormal test result would indicate a trend toward a therapeutic response to dopaminergic agents even with a SPECT-TRODAT imaging pattern diverse from that found in SNP [20, 34].

NMS in PD and other forms of Parkinsonism is considered critical clinical biomarkers. We found some studies correlating SPECT-TRODAT with the presence of NMS [10, 13, 14, 17, 30, 33, 35]. Rizzo and Plazzi [35] observed a high incidence of reduced DAT uptake in patients with RSBD, suggesting that these patients should be followed longitudinally to check for conversion to PD. Despite the differences between groups, no correlations were demonstrated in the present study between the presence or absence of NMS and SPECT-TRODAT results. An exception occurred for a correlation between the severity of the SPECT-TRODAT result and the presence of NMS in patients with DIP (rho = −0.451, *p*=0.052), not previously described.

The main limitation of our study is that it is a cross-sectional study and not a longitudinal study. We collected the result of a diagnostic conclusion after a few consultations and only one SPECT-TRODAT. We believe that this may stimulate other researchers to perform longitudinal studies seeking to clarify the correlation between the absence or presence of one or more NMS and severity in reducing DAT uptake in SPECT-TRODAT. Although perceptually compatible with other series, the small number of patients with some types of Parkinsonism may also have interfered with our analyses.

We were able with our study to suggest that SPECT-TRODAT examination may be helpful in the diagnostic aid of Parkinsonism, especially in differentiating PD and SP. As corroborated by Tolosa et al. [31], the DaTSCAN SPECT follow-up examination can be an aid in establishing a diagnosis more quickly and more assertively. Some studies have already proven that the examination reduces diagnostic uncertainties. Most patients are satisfied to know that the test result is compatible with their symptoms [37].

In conclusion, in our study, SPECT-TRODAT-1 was important to corroborate a diagnostic hypothesis, mainly to differentiate some types of Parkinsonism. This study added new data to suggest using SPECT-TRODAT as an adjuvant test in diagnosing patients with Parkinsonism (especially of DIP and VP). The correlation changes found between NMS and age of symptom onset and severity on SPECT-TRODAT-1 examination in patients with DIP suggest the need for future longitudinal studies in this group. The results obtained in this study with the brain SPECT with 99mTC-TRODAT-1 helped in the differential diagnosis of 153 patients with Parkinsonism.

The reduction in striatal DAT uptake on brain SPECT scan with 99mTC-TRODAT-1 was directly related to the severity of motor signs in the synucleinopathies, including PD, expressed by higher scores on the MDS-UPDRS-III scale. Regarding age at disease onset and symptoms duration, no correlation was found, except in the group of drug-induced Parkinsonism. The youngest patients in this group were the ones who showed the most significant reductions in striatal DAT uptake, something not yet reported in other studies. Brain SPECT with 99mTC-TRODAT-1 helped to differentiate the groups with predominantly unilateral clinical and imaging changes from those with bilateral, mainly between patients with synucleinopathies and Parkinson's disease compared with cases of secondary Parkinsonism. Despite low accuracy and specificity, SPECT-TRODAT might be necessary for differentiating Parkinson's disease and drug-induced Parkinsonism. Finally, no correlation was observed between the presence or absence of nonmotor signs (NMS) and SPECT-TRODAT results, except in the drug-induced Parkinsonism group. In this group, the presence of NMS resulted in tests with a more significant reduction in striatal DAT. This is a finding not yet described in the literature.

## Figures and Tables

**Table 1:** Clinical and epidemiological data of the groups with Parkinsonism.

Variables	Synucleinopathies (SNP)			*p* ^(SNP)^	Tauopathies (TP)		*p* ^(TP)^	*p* ^(TP × SNP)^	DIP (19)	*p* ^(DIP × SNP)^	*p* ^(DIP × PD)^	VP (13)	*p* ^(VP × SNP)^	*p* ^(VP × PD)^
	PD (93)	MSA (7)	LBD (16)		PSP (2)	CBD (3)								
Gender—female (%)	55 (59.1%)	5 (71.4)	8 (50%)	0.614	1 (50%)	0 (0%)	0.40	0.592	13 (68.4%)	0.650	0.607	9 (69.2%)	0.675	0.558

Average age (years)	65.09 ± 12.5	65.1 ± 10.4	72.8 ± 7.6	0.051	73.0 ± 5.7	63.3 ± 13.1	0.206	0.093	72.11 ± 10.74	0.017	0.012	75.62 ± 8.36	0.004	0.002

Duration of illness (years)	3.58 ± 4.86	1.38 ± 0.99	1.7 ± 1.26	0.159	1.5 ± 0.71	2.6 ± 2.15	0.273	0.141	1.34 ± 1.29	0.002	0.024	4.30 ± 4.42	0.060	0.307

Education (years)	9.39 ± 4.98	7.57 ± 5.26	6.81 ± 4.40	0.154	9.0 ± 7.07	7.0 ± 3.61	0.346	0.317	5.89 ± 4.07	0.032	0.002	6.08 ± 3.88	0.073	0.011

UPDRS	29.1 ± 11.9	32.7 ± 16.0	37.5 ± 16.4	0.097	48.0 ± 17.0	63.7 ± 8.4	0.123	0.00004	28.8 ± 11.7	0.183	0.470	31.8 ± 16.9	0.219	0.228

H&Y stage														
1	4 (4.3%)	0 (0%)	2 (12.5%)	0.001	0 (0.0%)	0 (0.0%)	0.528	0.001	0 (0.0%)	0.040	0.998	1 (7.7%)	0.002	0.044
2	51 (54.8%)	2 (28.6%)	2 (12.5%)		0 (0.0%)	0 (0.0%)			13 (68.4%)			1 (7.7%)		
3	34 (36.6%)	3 (42.9%)	10 (62.5%)		2 (100.0%)	1 (33.3%)			6 (31.6%)			6 (46.2%)		
4	4 (4.3%)	2 (28.6%)	2 (12.5%)		0 (0.0%)	(2) 66.7%			0 (0.0%)			5 (38.5%)		

Clinical presentation														
Mixed/classic	62 (66.7%)	2 (28.6%)	1 (6.25%)	0.0002	0 (0%)	0 (0%)	1	<0.00001	17 (89.45%)	0.002	0.055	0 (0%)	0.0002	0.0001
Akinetic-rigid form	31 (33.3)	5 (71.4%)	15 (93.8%)		2 (100%)	3 (100%)			2 (10.55%)			13 (100%)		

Nonmotor signs														
Depression	52 (55.9%)	3 (42.9%)	11 (68.8%)	0.736	0 (0%)	0 (0%)	0.876	0.076	12 (63.2%)	1	0.619	5 (38.5%)	0.999	0.254
Constipation	58 (62.37%)	5 (71.4%)	8 (50%)	0.546	1 (50%)	3 (100%)	0.40	0.550	9 (47.37%)	0.478	0.304	7 (53.85%)	0.690	0.763
RLS	32 (34.4%)	0 (0%)	2 (12.5%)	0.135	1 (50%)	2 (66.7%)	1	0.143	4 (21.05%)	0.141	0.295	1 (7.7%)	0.020	0.059
RSBD	68 (73.1%)	5 (71.4%)	12 (75.0%)	0.313	2 (100%)	2 (66.7%)	1	0.640	10 (52.6%)	0.362	0.298	6 (46.2%)	0.262	0.217
DRSBD	18.9 ± 34.39	4.29 ± 8.98	23.63 ± 41.36	0.768	24.0 ± 33.94	0	0.136	0.664	6.0 ± 16.85	0.738	0.054	16.62 ± 36.18	0.839	0.411

PD: Parkinson's disease, MSA: multiple system atrophy, LBD: Lewy body dementia, *p*, SNP: synucleinopathies, TP: tauopathies, PSP: progressive supranuclear palsy, CBD: cortico-basal degeneration, DIP: drug-induced Parkinsonism, VP: vascular Parkinsonism, RLS: restless legs syndrome, RSBD: REM sleep behavioral disorder, DRSBD: duration of REM sleep disorder. ^∗^*p*<0.05 indicated statistical significance.

**Table 2:** Correlations of the brain SPECT with ^99m^Tc-TRODAT-1 of the groups with Parkinsonism.

Variables	Synucleinopathies (SNP)			*p* ^*∗*^ (SNP)	*p* (PD × MSA)	*p* (PD × LBD)	Tauopathies (TP)		*p* (TP)	*p* (TP × SNP)	*p* (TP × PD)	DIP (19)	*p* (DIP × SNP)	*p* (DOP × PD)	VP (13)	*p* (VP × SNP)	*p* (VP × PD)
	PD (93)	MSA (7)	LBD (16)				PSP (2)	CBD (3)									
Regarding normality (%)	93 (100%)	7 (100%)	16 (100%)	1			2 (100%)	3 (100%)	1	1	1	11 (57.9%)	<0.0001	<0.0001	10 (77.0%)	<0.0001	0.0001
Abnormal exam																	
Severity of the alterations																	
Normal or zero	0 (0%)	0 (0%)	0 (0%)	0.199	0.209	0.814	0 (0%)	0 (0%)	0.764	0.685	0.863	8 (42.1%)	<0.0001	<0.0001	3 (23.1%)	<0.0001	<0.0001
Mild reduction or 1	1 (1.1%)	1 (14.3%)	0 (0%)				0 (0%)	0 (0%)				4 (21.1%)			2 (15.4%)		
Moderate reduction or 2	19 (20.4%)	1 (14.3%)	3 (18.7%)				1 (50%)	1 (33.3%)				7 (36.8%)			3 (23.1%)		
Accentuate reduction or 3	56 (60.2%)	4 (57.1%)	8 (50%)				0 (0%)	2 (66.7%)				0 (0%)			5 (38.5%)		
Severe reduction or 4	17 (18.3%)	1 (14.3%)	5 (31.3%)				1 (50%)	0 (0%)				0 (0%)			0 (0%)		
Symmetry of the abnormal (%)																	
Asymmetric	77 (82.6%)	6 (85.7%)	13 (81.3%)	0.999	1	0.999	1 (50%)	3 (100%)	0.400	0.974	0.713	8 (42.1%)	<0.0001	<0.0001	6 (46.2%)	<0.0001	<0.0001
Symmetric	16 (17.2%)	1 (14.3%)	3 (18.7%)				1 (50%)	0 (0%)				3 (15.8%)			4 (30.8%)		
Normal	0 (0%)	0 (0%)	0 (0%)				0 (0%)	0 (0%)				8 (42.1%)			3 (23.1%)		
Laterality symptoms																	
Unilateral	89 (95.7%)	6 (85.71%)	13 (81.25%)	0.051	0.309	0.063	1 (50%)	3 (100%)	0.400	0.036	0.105	14 (73.78%)	0.028	0.006	8 (61.53%)	0.002	0.001
Left	35 (37.6%)	1 (14.3%)	8 (50%)				0 (0%)	2 (66.7%)				5 (26.3%)			2 (15.4%)		
Right	54 (58.1%)	5 (71.4%)	5 (31.3%)				1 (50%)	1 (33.3%)				9 (47.4%)			6 (46.2%)		
Undefined—bilateral	4 (4.3%)	1 (14.3%)	3 (18.8%)				1 (50%)	0 (0%)				5 (26.3%)			5 (38.5%)		
Laterality-SPECT-TRODAT																	
Unilateral	76 (81.72%)	6 (87.71%)	13 (81.25%)	0.999	1	1	1 (50%)	3 (100%)	0.400	0.394	0.717	11 (57.9%)	0.227	0.033	6 (46.2%)	0.065	0.009
Left	43 (46.2%)	3 (42.9%)	7 (43.8%)				1 (50%)	0 (0%)				8 (42.1%)			5 (38.5%)		
Right	33 (35.5%)	3 (42.9%)	6 (37.5%)				0 (0%)	3 (100%)				3 (15.8%)			1 (7.7%)		
Undefined—bilateral	17 (18.3%)	1 (14.3%)	3 (18.8%)				1 (50%)	0 (0%)				8 (42.1%)			7 (53.8%)		
Laterality corresponding to the most affected side (%)																	
Yes	68 (73.18)	5 (71.42)	11 (68.75)	0.689 (sim × não) 0.041 (bilat × contra)	1 (sim × não) 0.306 (bilat × contra)	0.508 (sim × não) 0.052 (bilat × contra)	2 (100%)	2 (66.6%)	1 (sim × não) 1 (bilat × contra)	0.807 (sim × não) 0.059 (bilat × contra)	0.938 (sim × não) 0.254 (bilat × contra)	17 (89.5%) (89.47%)	0.566 (sim × não) <0.0001 (bilat × contra)	1 (sim × não) <0.0001 (bilat × contra)	11 (84.6%) (84.61%)	0.824 (sim × não) <0.0001 (bilat × contra)	0.508 (sim × não) <0.0001 (bilat × contra)
Contralateral	64 (69%)	4 (57.1%)	8 (50%)				1 (50%)	2 (66.66%)				6 (31.6%)			4 (30.86%)		
Bilateral	4 (4.3%)	1 (14.3%)	3 (18.75%)				1 (50%)	0 0%)				11 (58%)			7 (53.8%)		
No	25 (26.9%)	2 (28.6%)	2 (12.5%)				0 (0%)	1 (33%)				2 (10.5%)			2		
Ipsilateral	9 (9.7%)	1 (14.3%)	2 (12.5%)				0 (0%)	1 (33.33%)				1 (5.26%)			0 (0%)		
Undefined	16 (17.2%)	1 (14.3%)	0				0 (0%)	0 (0%)				1 (5.26%)			2 (15.4%)		

PD: Parkinson's disease, MSA: multiple system atrophy, LBD: Lewy body dementia, *p*, SNP: synucleinopathies, TP: tauopathies, PSP: progressive supranuclear palsy, CBD: cortico-basal degeneration, DIP: drug-induced Parkinsonism, VP: vascular Parkinsonism, RLS: restless legs syndrome, RSBD: REM sleep behavioral disorder, DRSBD: duration of REM sleep disorder. ^∗^^*∗*^*pp*<0.05 indicated statistical significance.

## Data Availability

Data are available on request.
